# Severe udder cleft dermatitis lesion transcriptomics points to an impaired skin barrier, defective wound repair and a dysregulated inflammatory response as key elements in the pathogenesis

**DOI:** 10.1371/journal.pone.0288347

**Published:** 2023-07-24

**Authors:** A. S. Vermeersch, M. Ali, Y. Gansemans, F. Van Nieuwerburgh, P. Geldhof, R. Ducatelle, D. Deforce, J. Callens, G. Opsomer

**Affiliations:** 1 Department of Internal Medicine, Reproduction and Population Medicine, Faculty of Veterinary Medicine, Ghent University, Merelbeke, Belgium; 2 Laboratory of Pharmaceutical Biotechnology, Faculty of Pharmaceutical Sciences, Ghent University, Ghent, Belgium; 3 Department of Translational Physiology, Infectiology and Public Health, Faculty of Veterinary Medicine, Ghent University, Merelbeke, Belgium; 4 Department of Pathobiology, Pharmacology and Zoological Medicine, Faculty of Veterinary Medicine, Ghent University, Merelbeke, Belgium; 5 Dierengezondheidszorg Vlaanderen, Torhout, Belgium; Université de Rouen Normandie, FRANCE

## Abstract

This study is the first to investigate the transcriptomic changes occurring in severe udder cleft dermatitis lesions (UCD) in Holstein-Friesian cows. An examination of the gene expression levels in natural UCD lesions and healthy udder skin through RNA Seq-Technology provided a deeper insight into the inflammatory pathways associated with this disease. A clear distinction between the gene expression patterns of UCD lesions and healthy skin was shown in the principal component analysis. Genes coding for inflammatory molecules were upregulated such as the chemokines C-X-C motif ligand 2 (*CXCL2*), 5 (*CXCL5*) and 8 (*CXCL8*), and C-C motif ligand 11 (*CCL11*). Moreover, the genes coding for the multifunctional molecules *ADAM12* and *SLPI* were amongst the highest upregulated ones, whereas the most downregulated genes included the ones coding for keratins and keratin-associated molecules. Predominantly inflammatory pathways such as the chemokine signaling, cytokine receptor interaction and IL-17 signaling pathway were significantly upregulated in the pathway analysis. These results point towards a fulminant, dysregulated inflammatory response concomitant with a disruption of the skin barrier integrity and a hampered wound repair mechanism in severe UCD lesions.

## Introduction

Udder cleft dermatitis (UCD) is a multifactorial disease that affects the skin between the udder halves and the front udder attachment [1, 2]. Lesions range from superficial papules and erythema to open, exudative wounds that pose a risk for debilitating conditions such as embolic pneumonia and udder vein pathologies [3]. Severe cases have the tendency to heal slowly when the skin integrity is breached and open wounds are formed. It can take up to 16–21 weeks in order to heal and even then cows are prone to relapsing [4, 5]. The within- and between-herd prevalence varies largely in literature, ranging from 0 to 44% [1, 4, 6]. There is no standard treatment or prevention of UCD.

A handful of studies have been conducted on the risk factors and eventual bacterial component in the etiology of the disease [1, 2, 4, 6–9]. Research points towards a shift in the microbiome with an accompanying loss of diversity [7–9]. So far, no specific pathogen(s) have been linked to the pathogenesis of UCD. Mange mites have been indicated as potential contributors by a few researchers [2, 8, 10]. However, a definite link with UCD lesions has not been demonstrated nor thoroughly examined. Bovine digital dermatitis has been linked to UCD lesions but a solid correlation could not be found [2, 6]. The presence of treponemes on udder skin has been confirmed in multiple studies through culture-independent methods, although their involvement remains to be proven [11–14]. Presence as such does not prove the status of treponemes as a definite causative pathogen since treponemes can also reside in slurry, feces, the oral cavity and other locations without causing any harm [15, 16]. The presence of bovine digital dermatitis-associated treponemes has been demonstrated in ischaemic teat necrosis lesions using 16S rRNA gene sequencing [17]. Mastitis-causing pathogens were not identified as potential contributors in multiple studies [7, 8]. Up to now, metagenomic analyses have not provided a possible explanation for the persistence of these painful, ulcerative udder skin lesions.

The existing literature lacks information on the host response to UCD. So far, to the best of our knowledge, no study has examined transcriptomic changes in these slowly healing skin lesions. Understanding the host response may, however, be indispensable when aiming to elucidate the full pathophysiology of these lesions. Therefore, the objective of the present study was to delve into the host response on a gene expression level with an emphasis on severe UCD lesions using high throughput RNA sequencing.

## Material and methods

### Sample collection and handling

The applied sampling protocol was approved by the ethical committee of the Faculty of Veterinary Medicine of Ghent University (dossier number 2021–103). Sample collection and processing were done in accordance with the relevant guidelines and regulations and all authors complied with the ARRIVE guidelines [18]. The animal health care center ’DGZ Vlaanderen’ sent a newsletter to Flemish veterinarians, in which a request for udder cleft dermatitis (UCD)-affected farms was put. Two Belgian dairy farmers located in Essen and Oostkerke voluntarily applied to participate in this study in 2022. The lesion samples were taken by a skilled veterinarian in small-average sized dairy farms with identical husbandry practices such as access to pasture and the presence of concrete slatted floors with cubicles. The sampled population consisted of adult, lactating cows of a variable age and parity. The farms were visited and in total 32 skin samples were taken from 16 different Holstein-Friesian cows displaying severe UCD lesions. According to the farmers, the animals had already been affected by UCD for several weeks but could not provide an exact timeline. Ten samples were taken from farm 1 with a herd size of 100 cows and the other six samples were taken from farm 2 with a farm size of 64 cows. The lesions were scored by the author as ‘severe’ due to the presence of large crusts, patches of thickened skin and an open wound prone to bleeding [2]. The body temperature was checked and the cows were inspected for the presence of lesions. Generally, the cows were in good health and did not have any other type of udder lesion besides the UCD lesion. Control samples were collected by the author from Holstein-Friesian cattle postmortem at the local slaughterhouse (Moeskroen, Belgium), after being clinically scored for the absence of any kind of udder (skin) lesion. Care was taken to only take samples from cows that did not have any external injuries. Sampling was restricted to a central location in the lesion. All biopsies were taken with a sterile, disposable 4 mm biopsy punch (Kai Medical, Solingen, Germany). Sixteen biopsies were directly put in a liquid nitrogen container after labeling (-196°C). The samples were stored in a -80°C freezer until further processing. The veterinarian remained in contact with the farmers to follow up on the sampled animals. For RNA isolation, the samples were put in a styrofoam container cooled by liquid nitrogen. The Qiagen RNeasy Minikit (Qiagen Benelux, Venlo, The Netherlands) was used in order to perform the RNA isolation in a RNase-free environment. The RNA quantity was checked using the NanoDrop 2000/2000c Spectrophotometer (Thermo Fisher Scientific, Geel, Belgium). Both the RNA quantity and quality were evaluated with the 2100 Bioanalyzer automated electrophoresis station using the RNA 6000 kit (Agilent Technologies Belgium S.A./N.V., Diegem, Belgium). The mean RNA integrity number (RIN) was 8.6 (s = 0.7) in lesion samples whereas the mean RIN was 6.9 (s = 0.3) in healthy tissue samples. Subsequently, the RNA was stored in a -80°C freezer before shipping the samples to NxtGent (Ghent University, Ghent, Belgium) for Illumina sequencing. In total, ten lesion and five control samples were selected based on having the best RNA quality and quantity.

### Histological examination

Sixteen lesion biopsies and five control samples were put in 10% neutral buffered formaldehyde and embedded in paraffin. Subsequently, 5 μm thick sections were cut. HE-staining was applied to the sections with the Varistain Gemini autostainer (Thermo Fisher Scientific, Geel, Belgium). A light microscope was used to assess the slides (Leica DM BD2, Leica Microsystems CMS GmBH, Mannheim, Germany).

### RNA sequencing

The QuantSeq 3’ mRNA-Seq Library Prep Kit FWD for Illumina (Lexogen Inc, Greenland, NH, USA) and the UMI Second Strand Synthesis Module for QuantSeq FWD (Illumina Inc, San Diego, CA, USA) were used to construct a sequencing library. The libraries were sequenced on a NextSeq device (Illumina Inc, San Diego, CA, USA) as a single-end 76. The unique molecular identifiers were removed from the raw reads with UMI-tools (v1.1.2). The quality and the length of the raw reads were inspected utilizing FastQC (v0.11.9) [19]. FastQ Screen (v0.15.0) [20] and a set of common lab organism genomes were used to check for putative contaminations. Up to 95% of the sequenced reads map on the bovine genome, whereas the non-bovine reads did not map to the tested lab organisms. The quality of the reads based on phred scores was good. Cutadapt (v3.7) [21] was used to trim the adaptors and filter reads containing ambiguities or reads that did not pass the phred score threshold of 20. Finally, the quality was checked with FastQc (v0.11.9) [19].

### Differential gene expression analysis

The splice-aware Spliced Transcripts Alignment to a Reference (STAR) mapper (v2.7.10a) [22] was used to map the processed reads to the bovine genome (ARS-UCD1.2). UMI-Tools (v1.1.2) [23] was used to deduplicate mapped reads. Rsem-calculate-expression (RSEM) (v1.3.0) [24] was used to count the features at the gene and transcript isoform level. The EdgeR package (v3.36) [25] in R (v4.1.2) was used for all statistical analyses. The filterByExpr() function discarded lowly expressed features. A principal component analysis (PCA) and scree plot of the rlog-transformed filtered data were made. To inspect the samples for outliers or labelling errors, PCA plots were generated in R, after transforming the raw gene counts with rlog() of the DESeq2 package to stabilize variances. Counts were normalized with the TMM method [25] and dispersions were estimated. The quasi likelihood model and F-test were used in order to perform statistical tests on the gene-level. The Benjamini-Hochberg method [26] was used to correct the p-values for multiple testing. Differential gene expression (DEG) analysis was done by a comparison between gene-level expression counts of healthy skin versus skin affected by severe UCD, using the edgeR package in R [25]. The gene fold changes obtained from the differential expression analysis were used to perform gene ontology (GO) term enrichment and pathway enrichment analysis in R with the GAGE package [27].

### qPCR analysis

To confirm the results of the RNA sequencing, three genes were selected for performing qPCR: interleukin-8 (*CXCL8*), disintegrase and metalloproteinase 12 (*ADAM12*) and interleukin-2 (*CXCL2*). The housekeeping genes *RPLP0* (60S acidic ribosomal protein P0) and *GAPDH* (glyceraldehyde-3-phosphate dehydrogenase) were chosen as the reference genes for the normalization of the data based on the normalization factors calculated in Genorm [28]. The delta Ct method was used to calculate relative quantities (Q values) to determine the fold changes in gene transcription levels. The UCD lesion samples were compared to healthy udder skin samples, leading to the mean fold changes in gene transcription levels. Primers were designed using PrimerBLAST, based on the bovine reference gene sequences from the National Centre for Biotechnology Information (NCBI) database. A BLAST of the primer sequences was done against the Refseq database of *Bos taurus*. The amplification reaction with SYBR Green Master Mix (Applied Biosystems, Ghent, Belgium) was carried out in the StepOnePlus Real-Time PCR System (Applied Biosystems, Ghent, Belgium). A negative control in the form of a no template control (NTC) was added to check for primer-dimer formation and contamination. The latter contained all RT-PCR reagents with the RNA template replaced by nuclease-free water. S3 File contains the characteristics of the primers used in the present study. The qPCR data was statistically analyzed with JASP software [29]. The nonparametric Mann Whitney U test detected the variations in gene expression. A p-value of <0.05 was considered to be significant.

## Results

### Histological examination

**Fig 1:**
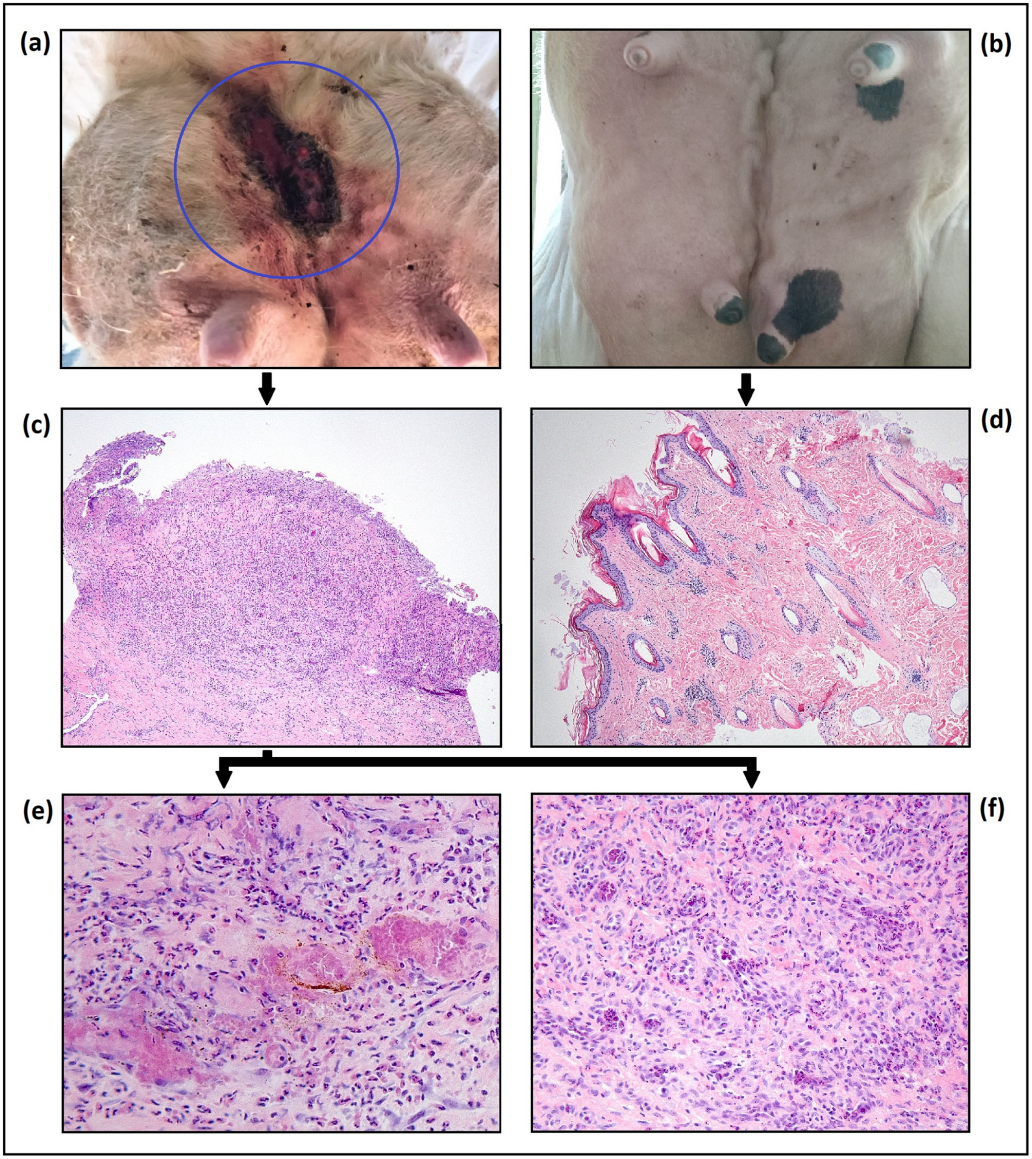
Macro-and microscopical display of a udder cleft dermatitis (UCD) lesion and healthy skin. The top row (a, b) shows a severe UCD lesion on the left and healthy skin on the right. The middle row (c, d) shows a histological view of a severe UCD lesion sample (magnification 50x) and a healthy skin biopsy (magnification 25x). On the bottom left image (e) a small hemorrhage can be seen and on both bottom pictures (e, f) a dense lymphoplasmocytic infiltration is present (magnification 200x). The separate images have been provided by AV. Dr. Piepers of Ghent University kindly provided picture b of this figure.

Fig 1 shows the results of the macroscopic and histological analyses. The hematoxylin-eosin (HE) staining was used to observe the characteristics of the skin tissue (thickness epidermis, cell infiltration and rete ridges). The majority of the histological severe udder cleft dermatitis (UCD) samples did not display a distinguishable epidermis due to the depth of the ulcerations. However, three UCD samples had a strongly thickened epidermis. Erythrocyte infiltrates and loss of epithelium were discernible. Moreover, dense lymphoplasmocytic infiltration was seen in the diseased samples.

### RNA sequencing

Libraries of a good quality with an average size of 16 million reads (S^2^ = 1.5) were generated (Table 1).

**Table 1:** Total number of trimmed sequences in millions generated with FastQC of udder cleft dermatitis samples and healthy skin control samples.

	UCD	Control
Sample 1	15.3	17.8
Sample 2	16.6	16.5
Sample 3	16.9	14.7
Sample 4	14.5	15.1
Sample 5	17.0	18.6
Sample 6	14.3	
Sample 7	18.9	
Sample 8	17.1	
Sample 9	14.7	
Sample 10	15.7	

**Fig 2:**
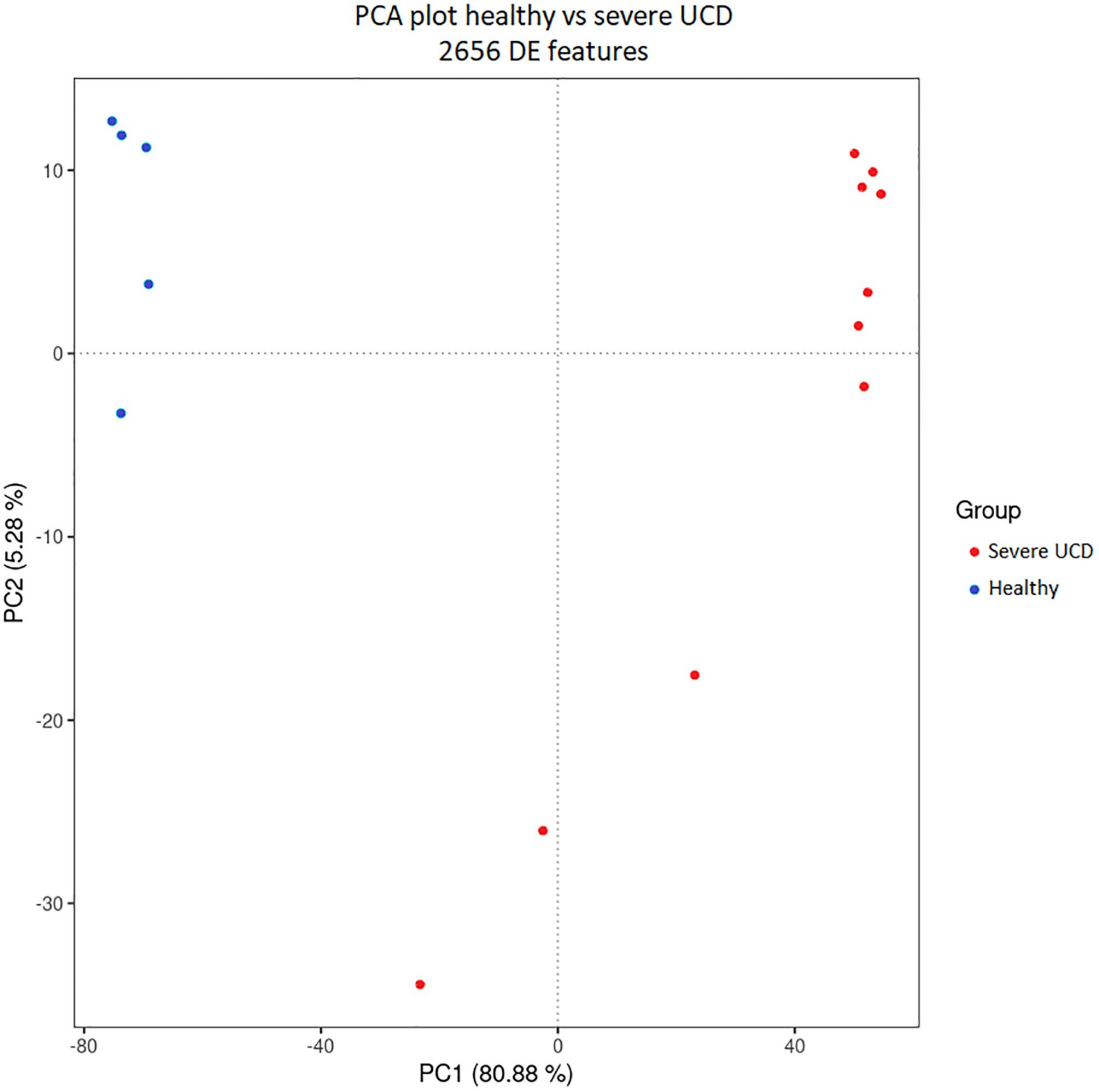
Principal component analysis (PCA) plot of the data. The replicates of the severe udder cleft dermatitis (UCD) lesions and the healthy udder skin are shown on the PCA plot. The ’regularized log’ (rlog) transformation of the raw gene counts was variance stabilized. Consequently the principal components were calculated. Figure made in R version 4.1.2 (https://www.r-project.org) supplied by YG and MA.

A principal component analysis was performed in order to further assess the quality and explore the generated data. The outcome of the analysis is visualized by a PCA plot shown in Fig 2. The formation of two distinct groups on the PCA plot can be noted, which reflects the expected biological differences between healthy and diseased tissue. The separate groups represent tissue from healthy cows versus from cows with severe UCD lesions. Three UCD samples do not group with the other UCD lesion samples. There is no biological nor technical reason that could explain this discrepancy. Subsequently, the differentially expressed genes were identified by comparing healthy skin samples with UCD samples. In total, 2656 genes were significantly differentially expressed, of which 930 genes were downregulated and 1726 gene were upregulated in the samples from diseased animals (adjusted p-value Padj ≤ 0.05). The ten most up-and downregulated genes can be found in Table 2 and the entire list of statistically significant DEG (Padj ≤ 0.05) can be found in S1 File. The fold change thresholds for the up-and downregulated genes were set at 2 and -2, respectively. Amongst the most downregulated genes, were those coding for keratins and keratin-associated proteins such as *KRTAP3-1*, *KRT86* and *KRT25*. The most upregulated genes comprised of chemokine-coding genes such as C-X-C motif ligand 2 (*CXCL2*), 5 (*CXCL5*) and 8 (*CXCL8*) and C-C motif ligand 11 (*CCL11*). Furthermore, the metalloproteinase encoding gene *ADAM12* was highly upregulated besides secretory leukocyte peptidase inhibitor (*SLPI*). In our dataset, the acute phase reactant serum amyloid A2 (SAA2) had the highest upregulation.

**Table 2:** The 10 most up- and downregulated differentially expressed genes in samples udder cleft dermatitis (UCD) lesions in comparison to healthy udder skin tissue.

Upregulated genes	Downregulated genes
*SAA2* (1328)	*MOGAT2* (-5865)
*ADAM12* (413)	*KRTAP3-1* (-3524)
*SLPI* (375)	*LOC785756* (-2744)
*PRND* (298)	*KRT85* (-2738)
*IL8* (254)	*KRT86* (-2692)
*CCL11* (223)	*KRT25* (-2192)
*ATP6AP1L* (220)	*ENSBTAG00000050955* (-1977)
*CXCL6* (214)	*PENK* (-1781)
*CXCL2* (195)	*SDR16C6* (-1706)
*SCN1A* (190)	*KRTAP11-1* (-1664)

The fold change is shown between brackets behind the gene designation (adjusted p-value Padj <0.05). Unknown proteins are noted with their ENSEMBL identity. The fold change threshold is set at ≥ 2 for upregulated genes and ≤ -2 for downregulated genes.

### Gene ontology enrichment analysis

Additionally, a gene ontology enrichment analysis was performed to functionally profile the DEGs. A total of 37 GO terms were statistically significantly overrepresented (Padj <0.05). These can be found in S2 File.

### Pathway analysis

Subsequently, a pathway analysis was performed in order to identify gene networks impacted in the UCD lesion samples. The analysis revealed 33 significantly upregulated and 1 downregulated pathway (Padj <0.05), of which the most significantly upregulated pathway was the chemokine signaling pathway. The IL-17 (Padj = 0.02) and the TNF signaling pathway (Padj = 0.02) were also prominently upregulated. The sole downregulated pathway was the ribosome pathway (Padj = 0.02). The pathways are included in S1 Table.

### qPCR analysis

The relative gene expression of *ADAM12*, *CXCL2* and *CXCL8* were significantly higher in UCD lesions compared to healthy udder skin samples (respectively p-value = 0.003; 0.006; <0.001). The outcome of the qPCR analysis is visualized with bar charts in S4 File.

## Discussion

In the current study the transcriptomic changes that occur in severe udder cleft dermatitis (UCD) lesions were analyzed. The principal component analysis displayed a significantly separate clustering of healthy versus UCD affected tissue. An upregulation in genes coding for molecules involved in the host innate immune response such as *SAA2*, *CXCL2*, *CXCL8* and *ADAM12*, and the wound healing mechanisms were revealed in the DEG analysis. Furthermore, a downregulation of genes mostly coding for keratins and keratin-associated proteins was unveiled. Pathway analysis indicated the activation of a plethora of pro-inflammatory pathways such as the chemokine signaling pathway, cytokine receptor interaction and the IL-17 signaling pathway. In a recent study, the IL-17 pathway has been identified as a consistently upregulated pathway throughout the acute and chronic stages of bovine digital dermatitis [30]. Both IL-17A and -F belong to the IL-17 cytokine family that helps to control infections but also plays a role in various chronic inflammatory conditions such as human psoriasis and rheumatoid arthritis [31, 32]. The activation of a neutrophil/Th17 cell-dependent immune response occurs through the upregulation of *CXCL8*, *Il-6*, *CCL20*, *IL-1β* and *G-CSF* [33, 34]. IL-17A also has a positive influence on tissue remodeling through the stimulation of metalloprotease secretion [35]. It leads to an increased proliferation and aberrant differentiation of keratinocytes and contributes to skin barrier disruption by downregulating the expression of molecules involved in keratinocyte differentiation such as filaggrin [36, 37]. Dysregulated IL-17A could prolong wound healing and promote inflammation [38–40]. This is in line with the macroscopical aspect of a severe UCD lesion, being a slowly healing wound. The transcriptomics of the severe UCD lesion do however not pinpoint one specific cause. However, an analogy can be drawn between UCD and canine intertrigo, where in the latter the lesions develop due to the combination of skin friction with local moisture and subsequent microbial overgrowth [41]. In the case of dogs the skin folds are mostly breed-and weight dependent. There is a lack of in-depth research on canine and human intertrigo, hence a comparison on gene-level is unfortunately not possible yet.

By far the highest upregulated gene was the one coding for the acute phase reactant serum amyloid A2 (*SAA2*) (adjusted p-value Padj = 0.002). A limited extrahepatic production occurs locally in the epidermis after exposure to stress e.g. to trauma, infection and inflammation [42, 43]. One of its many functions is to promote the chemotaxis of lymphocytes [44], monocytes [45] and polymorphonuclear leukocytes [45]. SAA also induces cytokines such as IL-1β, CXCL8 and TNFα [46], and stimulates the upregulation of MMP9 [47]. Besides its pro-inflammatory function, SAA is a prothrombotic mediator in atherogenesis and related diseases [48]. Overabundance of SAA2 could exacerbate the inflammation present in severe UCD wounds.

Concomitantly, a disintegrin and metalloproteinase 12 (*ADAM12*) gene was found to be significantly upregulated (Padj = 6.58E-05) in UCD lesions. In a study by Harsha et al. (2008), the expression of *ADAM12* was significantly higher in chronic ulcers in comparison to healthy skin [49]. An additional in vitro keratinocyte study showed an increase of keratinocyte migration in *ADAM12*-/- mice. The latter suggests that ADAM12 potentially plays a negative role in wound healing through reducing keratinocyte migration in the lesions. In a study by Liu et al. (2021), ADAM12 was almost exclusively expressed by T-cells, leading to an inflammatory response in tissue [50]. It has been proposed that ADAM12 plays a role in the regulation of the TGFβ signaling and Th17-cell differentiation in humans [51]. A study by Zhou et al. (2013) revealed that knocking down *ADAM12* in memory T-cells lead to an increased production of Th17 cytokines and enhanced the IL-17 secreting cell differentiation [51]. Contrastingly, in the present study both *ADAM12* and *IL-17* genes were upregulated. A future study on the protein expression would provide clarity in this matter.

The greatly upregulated *SLPI* has a number of diverse functions, ranging from anti-protease capacities for protection of matrix fibers, to anti-viral [52], anti-fungal [53] and anti-bacterial functions [54]. Dutch research indicated *SLPI* expression was highly upregulated in psoriatic skin and healing wounds [54]. Bactericidal effects have been demonstrated on *Pseudomonas aeruginosa* and *Staphylococcus aureus*, two skin bacteria with well-known pathogenic capacities. The authors hypothesize SLPI plays a role in the innate defense mechanism of the host skin, alongside the heavily upregulated cytokines.

Despite the chronicity of the severe UCD lesions, some early stage cytokines such as *IL-6*, *CXCL8* and *CXCL5* were significantly upregulated in our dataset. IL-6 is known to be involved during early inflammation and wound healing [55, 56]. Its expression is dysregulated in psoriatic skin or in case of a defective wound repair [55, 56]. CXCL8 and CXCL5 are an important part of the early post-injury neutrophil chemotaxis [57, 58]. Their presence in the longstanding and severe UCD-lesions hints towards a dysregulated inflammatory response.

An abundance of genes involved in wound healing, angiogenesis and tissue repair such as *HIF1α*, *S100A* and *integrin αv/β5*, and matrix metalloproteinases (*MMP*) 1/2/3/9/12/13/19 were significantly upregulated. The *MMP* functions are possibly dampened through the simultaneous upregulation of tissue inhibitor of metalloproteinase 2 (*TIMP2*) (Padj = 0.002). A dysregulation of the TIMP-MMP balance could lead to damage to the extracellular matrix. Besides *ADAM12* and *MMP*s, ADAMs with thrombospondin motif (*ADAMT*) were upregulated as well. These molecules have diverse roles in the different phases of wound healing. Wound healing is supposed to be an orchestrated series of events. However, transcriptional data of MMPs do not always accurately reflect the in vivo proteome due to possible inhibitors like TIMP. An excessive amount of MMPs, like for example MMP9 has the opposite effect as it potentially exacerbates chronic wound pathogenesis [59]. The early expression of MMP2 and MMP9 possibly helps to degrade biofilm matrix produced by wound invading bacteria [60]. Metallothioneins 2A and 4 appear to be downregulated in the present study (respectively Padj = 0.002; 1.35E-07). Through their zinc and copper binding properties, they potentially promote cell proliferation and re-epithelialization in skin wounds [61]. The combination of downregulated metallothioneins and excessive upregulation of various MMPs, likely leads to a hampered wound healing mechanism.

Some genes coding for keratins and keratin-associated molecules were heavily downregulated in the present dataset. Keratins have an important mechanical function and contribute to cell regulating functions such as differentiation, transport and signaling. They are a fundamental component of the epidermal architecture and offer protection against physical stress [62]. Keratins known to be associated with wound repair (KRT6, KRT16 and KRT17), were not presented with a significantly different gene expression in our dataset [63]. *KRT15* was significantly downregulated (Padj = 3.96E-07) which points towards activation of the keratinocytes [64]. On the whole, this downregulation leads to a disturbed barrier function of the epidermis, leading to a higher vulnerability of the skin for bacterial invasion and colonization [65]. Repeating the experiment with more samples and sample groups such as different breeds, lactation stages, housing and environmental factors could improve the generalizability of the research on UCD.

## Conclusion

This research provides the very first broad overview of the host response associated with severe udder cleft dermatitis (UCD) lesions in dairy cattle. The data point towards an overwhelming upregulation of matrix modifying MMPs and pro-inflammatory genes, potentially keeping the wound from healing properly. Furthermore a declined migration and aberrant differentiation of keratinocytes concomitant with a delay of re-epithelialization through e.g. *ADAM12* and *IL-17A* is likely. The disturbed keratinocyte function, continuous damage through matrix remodeling and inflammation, together with a damaged skin barrier integrity potentially conducts the formation of a chronic wound that is prone to infection. The data indicate a chronic and persistent, dysregulated inflammation and hampered skin healing accompanied by an impaired skin barrier. Our data does not lead straightly to a successful treatment option. Taking our novel findings into account, emphasis could be put on using products which dampen the heavy inflammatory response while concomitantly stimulate the keratinocyte and skin matrix function.

## Supporting information

S1 Table A pathway analysis revealed gene networks that were significantly impacted in the udder cleft dermatitis (UCD) lesions compared to healthy udder skin tissue. The adjusted p-value was set to <0.05.
(DOCX)

S1 File The entire list of statistically significant DEG (Padj ≤ 0.05) in severe UCD lesions. The tables contain the Ensembl ID, gene symbol, description, fold change (FC), p-value and the adjusted p-value (Padj). The fold change thresholds for the up-and downregulated genes were set to 2 and -2, respectively.
(XLSX)

S2 File Gene ontology enrichment analysis. Gene ontology enrichment analysis revealed statistically overrepresented GO terms in udder cleft dermatitis lesions (Padj < 0.05).
(XLSX)

S3 File The primer sequence, accession number and source of the primers used for the subset of five genes utilized in qPCR analysis of the given samples. (PDF)

S4 File A display of the relative gene expression levels in the different DD stages for ADAM12, CXCL2 and CXCL8. The data was obtained through qPCR analysis. The standard error is shown layered on top of the bars. The asterisk above a bar indicates there’s a significant difference between the relative gene expression from the healthy udder skin and the severe UCD lesion sample. The threshold of significance is set at Pval<0.05. (a) The expression of ADAM12 in the UCD samples is significantly different from the healthy udder skin samples. The p-value is 0.003. (b) The expression of CXCL2 in the UCD samples is significantly different from the healthy udder skin samples. The p-value is 0.006. (c) The expression of CXCL8 in the UCD samples is significantly different from the healthy udder skin samples. The p-value is <0.001.
(PDF)
