# Supplementary material for: Severe udder cleft dermatitis lesion transcriptomics points to an impaired skin barrier, defective wound repair and a dysregulated inflammatory response as key elements in the pathogenesis
Source: PLoS One. 2023 Jul 24;18(7):e0288347. doi: 10.1371/journal.pone.0288347 (PMC10365316; doi:10.1371/journal.pone.0288347)
Supplement: S1 Table — The adjusted p-value was set to <0.05. (DOCX) [file pone.0288347.s001.docx]

**S1 Table. A pathway analysis revealed gene networks that were significantly impacted in the udder cleft dermatitis (UCD) lesions compared to healthy udder skin tissue.**

| Upregulated pathways | Downregulated pathways |
| --- | --- |
| Chemokine signaling pathway (Padj = 0.0003)  Malaria (Padj = 0.0003)  Cytokine-cytokine receptor interaction (Padj = 0.0019)  Focal adhesion (Padj = 0.0081)  Pathways in cancer (Padj = 0.0161)  Relaxin signaling pathway (Padj = 0.0161)  Human cytomegalovirus infection (Padj = 0.0168)  PI3K-Akt signaling pathway (Padj = 0.0171)  Kaposi sarcoma-associated herpesvirus infection (Padj = 0.0171)  ECM-receptor interaction (Padj = 0.0171)  IL-17 signaling pathway (Padj = 0.0171)  TNF signaling pathway (Padj = 0.0171)  Pertussis (Padj = 0.0174)  Protein digestion and absorption (Padj = 0.0190)  Proteoglycans in cancer (Padj = 0.0190)  Amoebiasis (Padj = 0.0190)  Osteoclast differentiation (Padj = 0.0241)  Hematopoietic cell lineage (Padj = 0.0263)  AGE-RAGE signaling pathway in diabetic complications (Padj = 0.0276)  Rheumatoid arthritis (Padj = 0.0276)  Viral protein interaction with cytokine and cytokine receptor (Padj = 0.0276)  Platelet activation (Padj = 0.0276)  Complement and coagulation cascades (Padj = 0.0289)  Cell adhesion molecules (Padj = 0.0297)  Dilated cardiomyopathy (Padj = 0.0366)  Hypertrophic cardiomyopathy (Padj = 0.0366)  Apelin signaling pathway (Padj = 0.0366)  Rap1 signaling pathway (Padj = 0.0366)  Phospholipase D signaling pathway (Padj = 0.0428)  Leishmaniasis (Padj = 0.0429)  Chagas disease (Padj = 0.0479)  Circadian entrainment (Padj = 0.0483)  NOD-like receptor signaling pathway  (Padj = 0.0483) | Ribosome (Padj = 0.0240) |

The adjusted p-value was set to <0.05.
