# Supplementary material for: Severe udder cleft dermatitis lesion transcriptomics points to an impaired skin barrier, defective wound repair and a dysregulated inflammatory response as key elements in the pathogenesis
Source: PLoS One. 2023 Jul 24;18(7):e0288347. doi: 10.1371/journal.pone.0288347 (PMC10365316; doi:10.1371/journal.pone.0288347)
Supplement: S3 File — (PDF) [file pone.0288347.s004.pdf]

Supplementary file 3: The primer sequence, accession number and source of the primers used for the subset of five genes utilized in qPCR analysis of the given samples.

Gene	Primer sequence (5'-3')	GenBank Accession number	Source
**GADPH**	F: GGGTCATCATCTCTGCACCT<br>R: GGTCATAAGTCCCTCCACGA	NM_001034034.1	Refaai et al., 2013
**RPL0**	F: CTTCATTGTGGGAGCAGACA<br>R: GGCAACAGTTTCTCCAGAGC	NM_001012682.1	Refaai et al., 2013
**CXCL8**	F: GTTGCTCTCTTGGCAGCTTT<br>R: GGTGGAAAGGTGTGGAATGT	NM_173925.2	Refaai et al., 2013
**ADAM12**	F: CCGAGAGTTTCAGAGGCAAG<br>R: GGGTCCTGACTTATGGAGCA	NM_001001156	NCBI database
**CXCL2**	F: GCCACTCTCAAGACTGGTCA<br>R: GGGCAGGGTCTACTTCTGGA	NM_174299.3	Sipka et al. (2014)
