# Supplementary material for: Severe udder cleft dermatitis lesion transcriptomics points to an impaired skin barrier, defective wound repair and a dysregulated inflammatory response as key elements in the pathogenesis
Source: PLoS One. 2023 Jul 24;18(7):e0288347. doi: 10.1371/journal.pone.0288347 (PMC10365316; doi:10.1371/journal.pone.0288347)
Supplement: S4 File — The data was obtained through qPCR analysis. The standard error is shown layered on top of the bars. The asterisk above a bar indicates there’s a significant difference between the relative gene expression from the healthy udder skin and the severe UCD lesion sample. The threshold of significance is set at Pval<0.05. (a) The expression of ADAM12 in the UCD samples is significantly different from the healthy udder skin samples. The p-value is 0.003. (b) The expression of CXCL2 in the UCD samples is significantly different from the healthy udder skin samples. The p-value is 0.006. (c) The expression of CXCL8 in the UCD samples is significantly different from the healthy udder skin samples. The p-value is <0.001. (PDF) [file pone.0288347.s005.pdf]

Supplementary file 4: A display of the relative gene expression levels in the different DD stages for ADAM12, CXCL2 and CXCL8.

The data was obtained through qPCR analysis. The standard error is shown layered on top of the bars. The asterisk above a bar indicates there's a significant difference between the relative gene expression from the healthy udder skin and the severe UCD lesion sample. The threshold of significance is set at Pval<0.05.

(a) The expression of ADAM12 in the UCD samples is significantly different from the healthy udder skin samples. The p-value is 0.003.

(b) The expression of CXCL2 in the UCD samples is significantly different from the healthy udder skin samples. The p-value is 0.006.

(c) The expression of CXCL8 in the UCD samples is significantly different from the healthy udder skin samples. The p-value is <0.001.

(a)

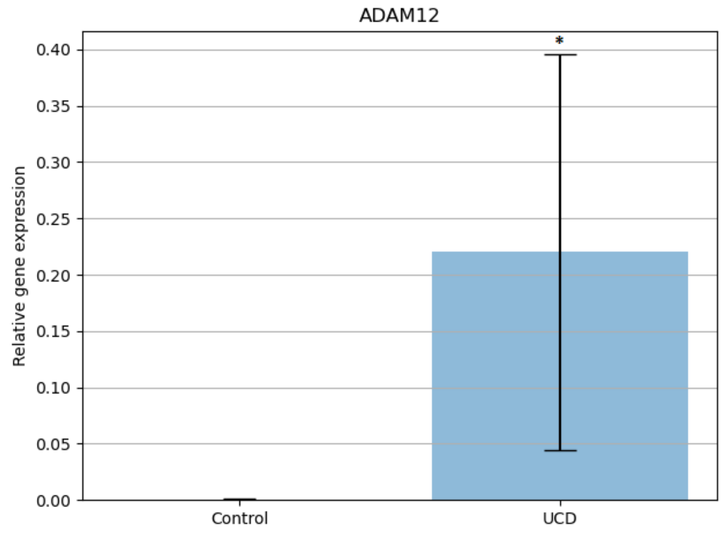
ADAM12
Relative gene expression
0.40
0.35
0.30
0.25
0.20
0.15
0.10
0.05
0.00
*
Control
UCD

(b)

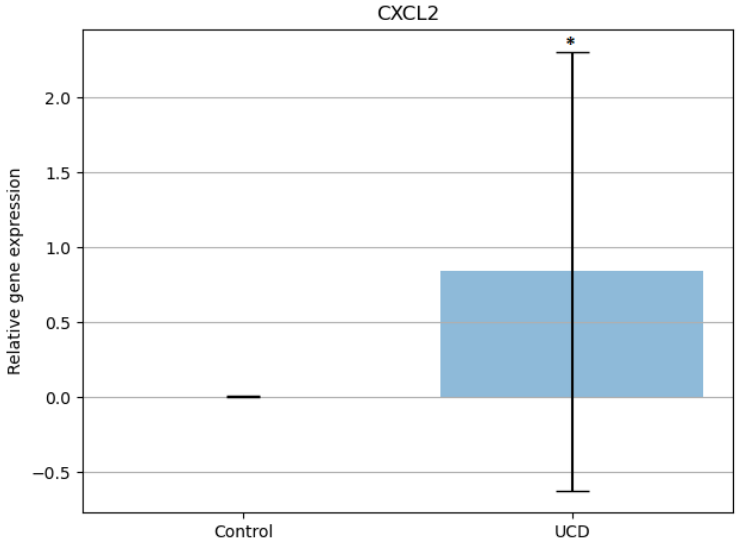
CXCL2
Relative gene expression
2.0
1.5
1.0
0.5
0.0
−0.5
*
Control
UCD

(c)

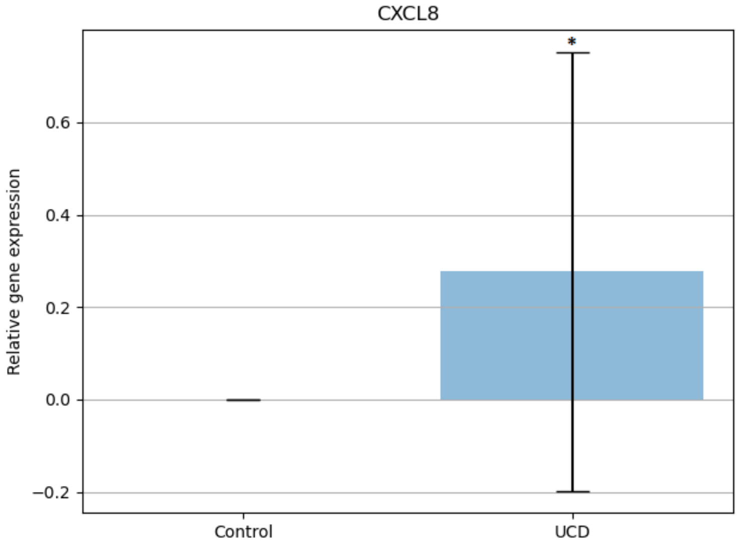
CXCL8
Relative gene expression
0.6
0.4
0.2
0.0
−0.2
*
Control
UCD
